# Retention of health workers in rural Sierra Leone: findings from life histories

**DOI:** 10.1186/s12960-016-0099-6

**Published:** 2016-02-01

**Authors:** Haja R. Wurie, Mohamed Samai, Sophie Witter

**Affiliations:** 1 ReBUILD Research Consortium, College of Medicine and Allied Health Sciences, Freetown, Sierra Leone; 2 Ministry of Health and Sanitation, Freetown, Sierra Leone; 3 ReBUILD/IIHD, Queen Margaret University, Edinburgh, Scotland

**Keywords:** Health workers, Sierra Leone, Retention, Distribution, Rural areas, Life histories

## Abstract

**Background:**

Sierra Leone has faced a shortage and maldistribution of staff in its post-conflict period. This long-standing challenge is now exacerbated by the systemic shock and damage wrought by Ebola. This study aimed to investigate the importance of different motivation factors in rural areas in Sierra Leone and thus to contribute to better decisions on financial and non-financial incentive packages, here and in similar contexts.

**Methods:**

This article is based on participatory life histories, conducted in 2013 with 23 health workers (doctors, nurses, midwives and Community Health Officers) in four regions of Sierra Leone who had worked in the sector since 2000. Although the interviews covered a wide range of themes, here we present findings on motivating and demotivating factors for staff, especially those in rural areas, based on thematic analysis of transcripts.

**Results:**

Rural health workers face particular challenges, some of which stem from the difficult terrain, which add to common disadvantages of rural living (poor social amenities, etc.). Poor working conditions, emotional and financial costs of separation from families, limited access to training, longer working hours (due to staff shortages) and the inability to earn from other sources make working in rural areas less attractive. Moreover, rules on rotation which should protect staff from being left too long in rural areas are not reported to be respected.

By contrast, poor management had more resonance in urban areas, with reports of poor delegation, favouritism and a lack of autonomy for staff. Tensions within the team over unclear roles and absenteeism are also significant demotivating factors in general.

**Conclusions:**

This study provides important policy-focused insights into motivation of health workers and can contribute towards building a resilient and responsive health system, incorporating the priorities and needs of health workers. Their voices and experiences should be taken into account as the post-Ebola landscape is shaped.

## Background

There is a growing need to strengthen health systems in developing countries to help provide universal health coverage for all. In addition, health systems have to be responsive and resilient especially in light of the ongoing Ebola outbreak in West Africa. The numerous challenges faced by human resources for health (HRH) can compromise the sustainability of the healthcare delivery system. One such challenge is having an inadequate, maldistributed health workforce with rural areas, where the need is greater, at a disadvantage, which undermines health-strengthening efforts [1–3]. Rural posting is often considered less desirable by health workers due to a number of factors. These postings are usually associated with increased workload and poor financial and non-financial incentives that put rural health workers at a disadvantage [4], which make retention of health workers equally challenging. Poor retention of health workers in some health facilities translates into staff shortages, increased workloads and stress levels and a demotivated workforce [3, 5–7]. The WHO report on increasing the attraction and retention of health workers in rural areas highlights the need for improved retention mechanisms [8]. Development of such policies on retention and motivation should incorporate the voices of health workers [9].

Sierra Leone underwent civil war from 1991 to 2002. The health system in post-conflict Sierra Leone can be described as fragile and plagued by having inadequate HRH, together with a history of low, irregular remuneration for health professionals. In addition, attracting and retaining health workers in rural areas, where two thirds of the population can be found [10, 11], especially areas that are hard to reach, has proven difficult, which has created a geographical imbalance in the distribution of health workers [12]. This creates unequal access to healthcare services as the services are not available where the needs and impact are most needed [13, 14]. A study reported that 90% of all of the surgeons in Sierra Leone are concentrated in the capital city Freetown, meaning rural areas of Sierra Leone are catered by only 10% of the surgeons available in-country [15]. Existing data from the HRH Directorate at the Ministry of Health in Sierra Leone suggests that the staff to population density for doctors in the Western Area (where the capital city Freetown is based) was 0.07 per 1000 of the population in 2005 and 0.12 per 1000 of the population in 2011. On the other hand, in Koinadugu, a rural and difficult to reach terrain, the density for doctors was less than more than half that of the Western Area in 2005 and 2011 (0.03 per 1000 of the population and 0.05 per 1000 of the population, respectively). Staff density figures for nurses also followed this regional disparity trend [16]. This maldistribution limits access to and quality of healthcare delivery.

The vision of the Ministry of Health and Sanitation (MOHS) in Sierra Leone is to have an adequate, well-managed and efficient system in place that will create a motivated human resources for health fully empowered to provide equitable access and distribution of services leading to a healthy and productive Sierra Leone by 2015 [17, 18]. Efforts should be made to actualise this vision especially in light of the setback caused by the 2014 Ebola outbreak, by further elucidating and addressing the barriers and challenges. This is the focus of this article.

Addressing the issue of attraction and retention of health workers in rural Sierra Leone is poorly informed by evidence. Current incentive packages for the attraction and retention of health workers involve mostly monetary attributes, but very little is known about the effectiveness of such packages and their sustainability. To date, no study has focused on the impact of decisions made, or not made, in post-conflict Sierra Leone on the attraction, retention, distribution and performance of health workers and thus ultimately on the performance of the health sector. This study aimed to investigate the importance of different motivation factors in rural, including hard-to-reach, areas in Sierra Leone and thus to contribute to better decisions on financial and non-financial incentive packages, here and in similar contexts.

## Methods

This is part of a HRH research project carried out by the ReBUILD consortium in Sierra Leone. The objectives of the overall study were:

1. To explore the overall perceptions and experiences of health workers before, during and after conflict
2. To identify their motivating and demotivating factors and coping mechanisms
3. To understand their views on the evolution of incentive policies for health workers post-conflict and factors which would encourage or discourage them from staying in post and being productive in remote areas
4. To derive recommendations for effective approaches, based on policy and what happens in practice, to retain health workers in hard-to-reach areas to support access to rational and equitable health services

The overall study included a health worker incentive survey, analysis of routine HR secondary data, stakeholder mapping, document review and key informant interviews, as well as the life histories which are the focus of this article.

The combination of methods was conceived so that each could build upon and triangulate with the others, within an overall framework which highlighted the linkages between context, policies and health worker features, and how these impact on human resource and health sector outcomes [19–21]. This article addresses objectives 2 and 4 above.

### Study design and tool

This substudy was qualitative, involving in-depth interviews with public health workers in urban and rural health facilities. A participatory life history tool [22, 23] was used to solicit their perceptions of their career in the pre-, during and post-conflict periods.

An open-topic guide was developed which covered the following topics:

1. How and why they became health workers
2. Their career path since they became health workers and what influenced it during and after the conflict
3. Overall career perception in terms of motivating and demotivating factors
4. Challenges they face in their job and coping strategies
5. Their career aspirations
6. Their knowledge and perceptions of incentive policies
7. Recommendation for an effective retention package for health workers in rural areas

Life lines were constructed to guide the discussion, as a useful prompt and cross-checking source during the interview, giving a summary of key events and decisions made over their working lives, as perceived by the health workers. This article is based on responses to topics 3 and 7.

### Site selection

Sierra Leone has four provinces—North, South, East and the Western Area, each subdivided into districts (14 in total). Four districts (Western Area (Urban/Rural), Kenema District (Eastern province), Bonthe District (Southern province) and Koinadugu District (Northern province)) were chosen as the study sites as being representative of the different provinces and including urban/rural variations and varying degrees of remoteness and poverty. Two of these districts are characterised as having very difficult terrains (Bonthe—riverine; Koinadugu—mountainous), lacking social amenities, electricity and running water supply. This makes working in these districts unattractive to health workers. On the other hand, Kenema and Western Area have large urban and rural populations and referral hospitals.

### Selection of study participants

A total of 23 in-depth interviews were conducted (see Table 1). It was intended that three health workers (one doctor, one nurse and one midwife) would be randomly selected from each district hospital of the study sites and three each from the main referral hospitals in the Western Area. In addition, two community health workers (Community Health Officers, CHOs) from each study district were to be interviewed—one more urban in location and one more remote. However, final numbers and distribution varied slightly according to availability of staff on the ground. Participants that had worked in the health sector since 2000 were selected, in order to capture the evolutionary theme of the study and understand how their lives had changed since the war. Due to this selection criterion, the participants were relatively senior cadres (all employed as public civil servants).

**Table 1:** What staff like and dislike about their jobs in order of frequency reported (comparing rural with urban respondents)

Motivating factors		Demotivating factors	
Urban	Rural	Urban	Rural
Being effective in their role	Community service	Difficult working conditions	Difficult working conditions
Financial incentives^a^	Being effective in their role	Limited financial incentives and benefits	Limited training opportunities and lack of career progression
Improved working conditions^a^	Financial incentives	Poor management (tensions in the workplace)	Limited financial incentives and benefits
Community service^a^	Training opportunities	Limited training opportunities and lack of career progression	Poor management (tensions in the workplace)
Training opportunities^a^	Religion	Political interference	Separation from family
		Strained relationships with the community^a^	Strained relationships with the community^a^
		Poor retention of staff	Insecurity^a^
			Political interference
			Poor retention of staff
			Challenges particular to rural postings, such as length of rural posting, lack of accommodation, high workload and long working hours

Summarised by researchers from in-depth interviews

^a^Same reported frequency in the grouping—i.e. urban or rural health workers

### Field work

The interviews were recorded by researchers, after gaining informed consent from the participants, and were conducted in a location selected by the respondent that they deemed as private and comfortable. Fieldwork was undertaken in March 2013 in the provincial study sites by an independent researcher (MM) and in October 2013 in the Western Area (by HW).

### Data analysis

The data was analysed from verbatim transcripts using the thematic framework approach, with the following stages: transcribing the interviews, familiarisation of the transcripts and the audio recordings, producing a coding framework (see Appendix), coding and identifying key themes from individual transcripts, merging themes, searching for key findings under each theme, comparing and finding associations, and providing explanations for the findings. The coding and analysis was led by a Sierra Leonean researcher (HW), with cross-checking and second reading by a UK-based researcher (SW). All transcripts were analysed looking for gender differences, differences in perception across the different cadres of health workers and urban versus rural differences.

This article analyses themes relating to motivation and rural retention. For analysis of the entire life history findings, see [24].

### Research ethics

Ethical approval was obtained from the Sierra Leone Scientific and Ethics Committee (25th May 2012) and the Liverpool School of Tropical Medicine prior to the commencement of the study. Informed consent was sought from the participants, assuring of confidentiality and anonymity of the information collected. There are ethical issues as regards reporting on interviews done with specific respondents who can be easily identified, e.g. where there is only one doctor per district or one monitoring and evaluation officer per district. To address this, findings were reported using codes without names or any details that would enable individuals to be easily identified.

## Results

Health workers were asked about their experiences of working and their recommendations for retention strategies in rural areas. Table 1 summarises the motivating and demotivating factors reported by participants in rural areas, using urban responses as a comparator. These factors are listed in order of the importance given to those findings by the participants. It is clear that rural and urban health workers have many shared motivating and demotivating factors but that their relative importance differs and that some are specific to rural areas. Factors of greater significance to rural posted health workers will be discussed further below. In the ‘Discussion’ section, we highlight how the rural perspectives differ from urban ones.

### Motivating factors

#### Community service

A sense of being of service and appreciation from within communities motivated health workers. There was a sense of communities holding health workers in high esteem as in some cases the health workers assumed other mentorship roles in society. In some instances, this motivated them to work around challenges or constraints, commit to long hours and improvise in difficult working environments to ensure that they continue to deliver service to the community, making a positive difference. For example, one respondent used his own initiative to encourage community members to utilise the health facility, ensuring that the best possible available healthcare service was sought.

…. well what I like most is when I see a patient walking into the hospital and going back with a smile and saying thank you going back home so I really love that and I appreciate that very much (Female, Koinadugu, IDI-9)

….because when I went there initially people were not making use of the facility […]so I said ‘No we must do something, let’s go into the community, motivate them.’ So we went […] we encourage them […] that is the time they started using the facility [….]Now today it’s one of the highest areas where pregnant women are giving birth [….] (Male, Western Area Rural, IDI-21)

#### Being effective in their role

Recognition in the workplace by their peers and feeling valued were key to this, as shown in the quote below in reference to a rural posting: ‘....but really I motivated so many people in that hospital….only 7 nurses were on payroll and then most of the people who were working at the hospital were volunteers. […].. so what I did I came back to Freetown and spoke with the personnel officer…. I think up to 58 people were appointed that year’ (Female, Western Area, IDI-13).

In addition, health workers were motivated by the acquisition of new skills that capacitated them to perform better in their jobs.

…because of some training we have learnt that and that is what we are also training other people to do. A lot, a lot of new ideas are coming which if we implement that it will be of great help (Male, Bonthe IDI-3)

#### Financial incentives

The health workers saw a decent salary that was paid on time as positive as it translated into being able to provide for their families.

I want to have a decent salary that will enable me to plan the lives of my children so that they too can be in the position to be of use to their communities in the future. (Male, Kenema, IDI-4)

……yes if I’m motivated, it will improve my livelihood because if salaries and remunerations are being paid on time […]and is increased ,that means I will satisfy my children even if I am away […]in paying the college fees and school fees for my children (Female, Koinadugu, IDI-9)

#### Training opportunities

Continued professional development in the form of short courses or on-the-job training was a motivating factor as it was seen to help with their career progression path.

even to improve our capacity in form of training I would appreciate that very much […] it’s serves as a motivation to me […] I was really happy ‘cause I thought at least I’ve had something in my CV (Female, Koinadugu, IDI-9)

#### Religion

Religion also emerged as a motivating factor, reinforcing empathetic characteristics for some:

…… I don’t want to be religious, but some of us as Christians, you know Christ was healing people, the great physician, and if I do delivery and the baby survives, anytime I see that baby going about, growing up I feel proud.,….. (Male, Bonthe, IDI-3)

### Demotivating factors

#### Difficult working conditions

The most frequently reported demotivating factor reported was poor working conditions, of which the majority of the respondents were in rural postings. Health facilities in these difficult terrains are especially understaffed, meaning additional work burdens on those who stay. The difficult mountainous terrain of Koinadugu and the riverine terrain in Bonthe and the lack of transport create challenges for health workers and service users alike.

Rural health facilities in particular were described as unhygienic as far back as the pre-conflict period and not conducive for effective work.

….. where we were having the clinic […]was not conducive for the work, the building was infested with rats, […]and we were all living in that building […]Water was not available […] For all the 5years I was there, I spent in that dilapidated building; it’s heavily infested with rats. (Male, Kenema, IDI-4)

Transport was also lacking, which created an additional burden for health workers posted to rural areas, and even more so for those in hard-to-reach areas. Ambulances were not provided to some health facilities and, coupled with poor road networks, created a barrier in trying to access patients at the primary health units (PHUs).

… there are some other people working in locations they are not mobile, they have to work over long distances, they have to pay transport (Male, Kenema, IDI-4)

#### Limited training opportunities

Regional disparities in the training and career progression opportunities influenced health workers’ decision to take up rural postings as captured in this study. These health workers reported the importance of the relationship between continued professional development and career progression in motivating them in the workplace.

…. I don’t have opportunity […] whenever there is an opportunity, to go for further course, we are not remembered. Everything is staying in Freetown […] And we are here. Are we not part of the nurses, are we not part of you people? Please try and think of us (Female, Bonthe, IDI-1)

Disparities in training opportunities based on type of health facility were also identified in this study. Health workers reported more training opportunities in primary health units compared to secondary and tertiary hospitals, resulting in them preferring to work in the former to have access to the training.

…I came from the primary healthcare now to the tertiary feeling that things would change […] I observed there are little trainings, little encouragement […] I observed that the little trainings that I was getting in the primary healthcare was not there [..] in secondary healthcare as it in primary health care, so I made up my mind that I should leave there back and go back to primary healthcare (Male, Koinadugu, IDI-12

Health workers working in the capital, Freetown, also reported that there was a lack of transparency in available training opportunities. Training opportunities and scholarships are not made available equally to all health workers.

… There will be people that are not even qualified to go for the training, they send them (Female, Western Area, IDI-15)

#### Lack of career progression

Community Health Officers (CHOs) were also dissatisfied about the lack of a career progression and promotion path in their profession and felt having a governing body was necessary. This would also in part address the high attrition of CHOs leaving government health facilities to work for NGO health facilities, which offered better work and pay conditions.

We don’t even have an Act to go to govern us, we don’t even know if we can be promoted or not, we don’t even know if there is any future in this career, so it’s disheartening (Male, Kenema, IDI-4)

This causing people to leave the Government to seek employment with other NGOs […] You do a good job but you are not compensated for that (Male, Bonthe, IDI-2)

#### Limited financial incentives and benefits

The study participants reiterated the historical low remuneration of health workers as a demotivating factor. This issue was addressed by the Government of Sierra Leone (GOSL) with the salary uplift that came with the Free Healthcare Initiative (FHCI) in April 2010. However, the general consensus was that the salary uplift was not in line with the increased cost of living and not commensurate with the duties health workers perform. The current salary provided by the GOSL was described as a ‘pittance’ and as ‘not encouraging’, especially when compared to what those working in the private sector (e.g. NGOs) earned.

…Well looking at the salary is it just a pittance (Male, Kenema, IDI-7)

In general, it was common for health workers to need to take a second job to supplement their income (e.g. engaging in petty trade), making them overworked and not fully committed to their health professional duties. In addition, financial incentives, such as the remote area allowance (RAA), introduced to motivate health workers to stay in rural postings, were described as not forthcoming. This demotivated rural health workers as they see no difference in their financial benefits compared to those working in urban areas.

…rural allowance right and we are not seeing none of these things on our salaries we do not see the differences between this and our colleagues elsewhere right and we are we are living in very hard to reach areas which are not easily even navigable by even motorbikes… (Male, Koinadugu, IDI-13)

#### Poor management and tensions in the workplace

Professional relationships between supervisors and those they manage, and between different cadres of health professionals (e.g. doctors and nurses), emerged as a demotivating factor in this study. Junior staff members felt neglected and unappreciated by those above them, creating conflict and division in the workplace. The high frequency of this theme as a demotivating factor indicates the important role of effective management for the motivation of health workers, especially in urban areas.

….. Some bosses may tend to be humiliating to others. Instead of them motivating you to work they tend to be humiliating for one reason or the other […]you allow somebody to hold everybody to ransom […] nobody can work, nobody can say anything, because you are the boss (Male, Western Area, IDI-21)

The respondents also expressed their disappointment in the lack of support from the central government and from their supervisors on behalf of health workers, meaning that factors affecting health worker motivation are not identified or addressed.

….but what I don’t like about the job is that we who are the cadre that is down there, who are seeing the bulk of these patients […] we feel abandoned. That is the funny thing about the Ministry of Health, it is the expectation that if we raise issues that are bothering us in our health facilities […]those people who are our supervisors must be taking these things forward and agitating so that these things will come, but this this does not take place (Male, Koinadugu, IDI-12)

Health workers in this study also felt that they should be involved in the decision-making processes that govern the management of the health facilities. There was no delegation of duties or teamwork, with doctors taking on senior managerial roles, including the finances, without the involvement of the other health professionals, even those in supervisory positions in secondary facilities, creating a managerial situation described as a ‘dictatorship’.

Other factors which are reported to cause tension and demotivation in the workplace included lack of punctuality, absenteeism and staff not working within professional remits. These can be perceived as the effects of a number of other factors, such as poor management. Lower cadre staff are having to take on roles they are not qualified to perform, unsupervised, which can be seen as cultivating indiscipline amongst staff.

And then you walk in to the ward situation an SECHN [as in state enrolled community health nurse] in charge. So I think you just kind of neutralize power, so it means there is not much control because when one SECHNs tells the other SECHN what to do.[…] So you find because of these gaps in the hierarchy there was not much control (Male, Kenema, IDI-6)

Issues of working with other lower cadres of health workers, such as the Maternal and Child Health (MCH) aides, and community-based workers, such as traditional birth attendants, were also raised. These two classes of health workers are being encouraged to work together with the main health workforce to improve health outcome and reduce child and maternal deaths, especially in the rural areas. However, this sometimes raises tensions about professional roles and hierarchies.

….some (referring to TBAs) who are still stubborn but because they have been educated that you bring the patients to the hospital they have been doing it and things are getting better because there are times when you have a lot of deaths (Female, Western Area, IDI-14)

…they are fussy but they think they are also taught and so they should not refer to the MCH aides, we have some of them who are still stubborn they think they can manage every case (Female, Western Area, IDI-14)

#### Separation from family

Separation from their families was cited as a demotivating factor by rural health workers and female health workers in particular in rural postings. Limited communication systems in the rural areas of Sierra Leone and infrequent visits home due to tight work schedules and travelling difficulties make it difficult for separated families to stay in touch, which causes emotional stress.

[…] being in the provinces so long, you are away from your family it has created some sort of social isolation and stress. Any way it is too much (Female, Koinadugu, IDI-9)

…..the distance is far from my family, my family is in Freetown. I have even a little girl who will be four in this month. I left her at one year plus […]I miss them a lot, they are growing without me […] So that is making it a little bit difficult [……] and my husband is complaining because he cannot come. He is like the mother and father so that is the problem (Female, Koinadugu, IDI-8)

#### Insecurity

Issues pertaining to personal security were experienced predominately by those working in hard-to-reach areas of Sierra Leone and especially by women, having to use inappropriate and sometimes risky means of transportation, such as overcrowded public minibuses.

….if I want to go to Freetown, I have to go out and use commercial vehicle. Like here if you don’t get up by 12/1am you will not get a vehicle to go to Freetown..[…]…and I have no alternative unless I decide to take a motor cycle. At my age on a motor cycle I don’t think it is appropriate […]….Did you hear about the death of our colleagues? […]If that lady had a vehicle or a vehicle was available for her to use within the DHMT, I don’t think she would have risked her life using a commercial vehicle. (Female, Bonthe, IDI-1)

#### Strained relationships with the community

Health workers reported instances of community members ‘policing’ them in the workplace and called for clearly defined roles and responsibilities in the community-monitoring scheme, which was introduced as part of the Free Healthcare civil society monitoring:

….. Well this monitoring, this community involvement ..[..]… I want it to be minimised because they are not here to police us [..] you see sometimes it is more demotivating … (Female, Kenema, IDI-9)

#### Political interference

Political interference, from those high up in society (e.g. politicians and top civil servants), described as minimal pre-conflict, but very common in the present, was reported across the board. Health workers are subjected to political interference at different stages of their professional lives, ranging from securing scholarships for further studies and promotions to posting and recruitment of health workers. Job postings are said to be changed due to political interference and deserving health workers are overlooked for promotion as only those with political backing are promoted. Health workers with political connections were reported to not adhere to disciplinary actions, which further fuels a sense of indiscipline in the work place.

Well now when a nurse goes out the way [is unprofessional], you want to discipline that nurse, you get order from above, whether you like it or not; […] interference, seniors are not allowed to do their work, the chain of command is lacking, there is no stand of control. (Female, Kenema, IDI-9)

#### Problems with the recruitment process

A number of issues feed into poor retention of health workers, ranging from high attrition of the available HRH to non-compliance with rural postings. This leads to increased workloads and in some instances unqualified staff working in roles that require more experience. Health workers expressed their dissatisfaction about the recruitment process into the health sector, which is currently controlled at the central level and does not involve senior local cadres who have to work with the recruited staff.

……. let me start first the recruitment process, because we are not responsible for training and recruiting some of the nurses and even the health workers, so that is a problem. (Female, Koinadugu, IDI-9)

Incidences of malpractice were also reported in the recruitment process, such as having to pay bribes to get applications reviewed more swiftly.

#### Rural postings

It was reported that the posting policy was not adhered to. Health workers are meant to be rotated after 2 years of a rural posting. A female health worker reported working at the same station for 10 years, with no fringe benefits, making her feel isolated, abandoned and inferior compared to her colleagues. With other health workers allowed to not take up rural posting due to political interference or no disciplinary measures for those who default, this is a demotivating factor. The transition to working in rural areas is also not smooth; accommodation is lacking for those posted to rural areas, and in some cases, good schools to continue with their children’s education are not available.

Because I have stayed longer up the provinces because there was a policy that you should just stay for two terms, but now, it has been long overdue. I have stayed for 10 years you see..[…].. of my colleagues [ those in urban areas] they are up there today [ have progressed along their career pathway] (Female, Kenema, IDI-9)

#### Long working hours

Health workers report being posted to the provinces to assume leadership roles in health facilities and having to cope with volunteers and untrained staff, rather than trained health personnel. It is a common occurrence for newly recruited staff posted to these difficult terrains not to report for duty, which increases the workload of health workers already in post, especially those in supervisory roles. This translates into health workers having to put in very long hours to maintain standards of service delivery and to offer technical assistance and supervise the untrained staff.

……I wouldn’t mind people knocking at my door at night because maybe people who are on night duty have only maybe one midwife and some cases she would not be able to cope and she cannot leave the volunteers to do anything because they are dangerous (Female, Western Area, IDI-14)

### Staff recommendations for a retention package for health workers

Creating a conducive working environment with improved health facilities, improved conditions of service, provision of decent staff accommodation or housing allowance, equal training opportunities, transportation allowance or transportation provided, improved salary scales, recruitment of more staff and regularisation of allowances pertaining to health workers in rural postings were recommended in order to recruit and retain health workers in rural postings.

Other recommendations include the establishment of a health workers’ commission, tasked with coordinating the affairs of health workers. A performance management system should also be introduced ensuring that the right level of professional support is being received. This would also minimise the attrition of senior experienced health workers, leaving junior recruits to manage the healthcare delivered in the health facilities unsupervised in some instances. In addition, the heavily centralised recruitment process should be reviewed to avoid inordinate delays and deter the occurrence of political interference. Most importantly, measures should be put in place to ensure the sustainability of the recommendations listed below.

#### Financial incentives

The most frequently reported recommendation was that salary scales should be increased and all allowances [RAA and performance based financing (PBF)] should be regularised. This will serve as compensation for health workers not being able to engage in extra jobs to generate extra income, a common occurrence in rural Sierra Leone which has negative implications for health workers’ commitment.

… salary should be based on the economic status of the day […] remote allowances meant for these health workers working in remote areas MUST be given to them on time and at the right time […]because when somebody works for something they should receive it immediately (Male, Bonthe, IDI-2)

because when I was in Freetown I was working in two places, but for now everything is cut off, just my bare salary and I have my family in Freetown, I have to support them, so it is not easy. That is why we working up in the provinces, we should be motivated. Even the salary scale should not be the same to be sincere (Female, Koinadugu, IDI-10)

Another recommendation was for the Government to introduce a loan scheme for health workers wherein goods and services can be easily accessed by health workers.

they should give us the right to go and take loan from government or from banks or from institutions to build our capacity… (Male, Koinadugu, IDI-11)

#### Accommodation

Another major deterrent in the retention of health workers in rural postings is the lack of suitable accommodation. Health workers reporting to the health facilities and leaving soon after due to lack of accommodation was reported as a common occurrence.

In fact I know a Matron who is having housing problems now and she is always grumbling ‘I am going back to Freetown because I cannot stay there I don’t have somewhere to stay, comfortable place’ so housing is the problem it is the number one problem (Female, Western Area, IDI-14)

Health workers also recommended that accommodation provided should be in close proximity to the health facilities for safety reasons.

Even for our housing facilities they should provide more quarters or if the quarters are not enough they should rent houses for nurses because most of us we should not be far away from the hospital […] it will not be easy for me get up from my bed far away to come to this place [as in the health facility]when I am afraid, because nobody is going to accompany me, no vehicle will be sent to go and collect me (Female, Koinadugu, IDI-10)

It was also recommended that housing allowance should be paid to health workers not provided with staff accommodation.

For staffs who are not housed in government quarters or community structures, accommodation allowances need to be paid to them (Male, Kenema, IDI-5)

#### Mobility and communication

Communication is a challenge due to lack of adequate mobile phone coverage. Health workers reported having to personally fund transportation in and around their working perimeter due to lack of transport logistics in the health facilities. In addition, they have to fund attending workshops in Freetown, as required by the job, from their salaries.

First you have to think of their transportation, you have to think of the remoteness of the place […] There are some of my colleagues who can pay not less than 100,000 Leones [approximately £15/$23], to come one way…[…]…and this is coming from your pocket. (Male, Bonthe, IDI-3)

For safety reasons particularly for female health workers, transportation should be made available in the instance that they are needed outside of working hours. An ambulance should also be provided for the health facilities ensuring that patients needing medical care are transported to the health facilities in a safe and controlled manner.

…Kenema now we have so many new areas that are not very ‘motorable’ […] so if we have transport collecting them and at point and taking them back after work […] we need people to work in the operating theatres but they are living far away […]So we begin to think now can we provide accommodation for them within the periphery of the hospital so they are just a stone’s throw from the hospital because these are all the things that can make the staff always readily available (Male, Kenema, IDI-6 )

It was also recommended that the Government should provide transportation for newly posted health workers to move to their posted locations as otherwise health workers have to arrange their personal transportation, which can be expensive and time consuming. This would also partly address the issue of health workers not reporting for duty in rural areas.

… whenever we are transferred […] they should provide with vehicle to come with our belongings, for it is not easy for us; maybe we take about two to three trips going to Freetown before we will be able to transfer all our belongings to this place (Female, Koinadugu, IDI10)

#### Training opportunities and career progression

Health workers in rural areas felt disadvantaged with regard to training opportunities. Therefore, the issue of regional disparities should be addressed to ensure health workers across the board have equal access to training opportunities. It was also reported that training opportunities are not made equally available even within the same region, with health workers in rural hospitals reporting that training opportunities in the PHUs are more compared to those available in the hospitals.

…we should be motivated by sending some of us to go and study, giving us scholarships. But what we find out most of us who are working up in this, in the provinces we are neglected […] MOHS when they say they have scholarship for people to go and do short term courses […] they should not only consider the Western Area.. (Female, Koinadugu, IDI-10)

It was recommended that training opportunities should be prioritised; low levels of staff working in the communities should be encouraged to gain further education and mid-level cadre health professionals, such as CHOs and staff nurses and midwives, should be trained to act as first-response units where higher cadres are not readily available. These measures will help facilitate career progression.

We want to be trained specially, we have CHOs that could want to be a paediatrics assistants, we have CHOs who want to be medical assistants, physicians, we want CHOs to be surgeon assistants, so where the doctors cannot go the CHO can go there and do it at community level, that is what we are yearning for (Male, Koinadugu, IDI-11)

#### Improvement of working conditions

It was reported that facilities have been neglected and have therefore created non-conducive working environments. Thus, it is recommended that they should be improved on in terms of the availability of drugs and functional equipment. Health facilities should be adequately staffed, which will have a positive impact on health workers’ workload and ultimately their working conditions; it was reported that some PHUs in rural areas only have one health professional working there.

Improve the facility I think that would be one […] the hospital environment should be clean, the wards or the wards should not be overcrowded..[…]…so that the patient have proper nursing care you […] the staff there should be of adequate number of staffs that I should be able to work with, I should not be deprived ‘cause I cannot work alone (Male, Western Area, IDI-18)

#### Social amenities

Provision of social amenities (satellite TV, internet services and electricity) will serve as an additional incentive for health workers in rural areas and making them feel less disconnected from urbanisation.

… provided with decent quarters, decent healthcare centres, mobility, if possible electricity, […] modern gadget like internet facilities or you can even have multi TV units [as in satellite TV] […] you can be touch with what is happening all over the world (Male, Koinadugu, IDI-4)

#### Separation allowance and relocation package

The introduction of ‘separation allowance’ should also be considered.

….and this is something I was trying to propagate that if you are going to send people out they need to have that separation allowance; because its 90% of nurses are females and most of them have families apart from the fact that they have to run two homes, you need to give them a separation allowance (Female, Western Area, IDI-23)

Some health workers would like to relocate with their families, but a number of factors, such as access to school for their children, should be addressed to make the transition easier. It was also recommended that the government should also consider providing financial support to health workers in rural postings for the first 6 months of the posting.

#### Transparent and fair procedures

The posting procedure should also be transparent with no political influence or otherwise with no disparities.

….let’s make it [posting procedure] transparent and equal opportunities, that is to say if we know for sure that as a policy you send one nurse to one rural area, this nurse works for 2 years in that particular posting she has completed her posting and then she knows when she goes to Freetown somebody else will be going and it should be fair and it should cut across (Male, Kenema, IDI-6)

## Discussion

This study explored motivational issues faced by health workers and their recommendations to improve retention in the rural areas of Sierra Leone. The recommendations relate to all of the factors identified as motivators and demotivators, with the exception of strained relations with communities, which are inherently harder to address through direct policy interventions. Addressing the recommendations in a coherent way should therefore improve motivation and retention within the thinly staffed rural areas of the country. Poor workers’ motivation can greatly affect health worker performance, health outcomes and patient safety [25]. There were several common motivational themes identified in this study that were consistent with findings from other studies in developing countries [3, 26–33] and in line with the WHO global policy recommendations on attraction and retention of health workers [8]. It is well documented in the literature that financial incentives and non-financial incentives are both of importance to health workers [2, 30, 34, 35].

In terms of study limitations, the sample had to be altered slightly in the light of actual staffing availability in the study sites and also the limited time available for higher level cadres, such as doctors. It also included health workers who stayed after the conflict, thus not including the viewpoint of those who had left. However, the life history tool employed allowed for detailed and contextualised probing and so for rich findings, which are complimented by other research methods [19].

This study showed that in Sierra Leone, the motivational factors most valued by the rural health workers were serving the community, being effective in your role followed by financial incentives, opportunities for on-the-job training and continued professional development and religion. This is compatible with the results of an open question in health worker survey carried out by ReBUILD on 312 health workers in Sierra Leone and with other related studies recently conducted in the region [21] Witter et al. Delivery fee exemption and subsidy policies: how have they affected health staff? Findings from a four-country evaluation, submitted. When asked to list and rank the main factors that motivated them to stay in their jobs, salary emerged as the most highly ranked in general, followed by opportunities for training and additional allowances/opportunities to serve the community. In addition, visible improvements in working conditions were another motivational factor expressed only by health workers in urban settings. This could be due to a number of reasons, including that in general rural health facilities (apart from those supported by an external agency) have seen less investment and hence less improvement in this aspect compared to urban health facilities.

### Differences across and within cadres

There was not much variation in what health workers reported as motivational factors across the different cadres of health workers captured in this study. The most reported motivational factor across the board by different cadres of health workers was financial incentives. Community service as a motivational factor was mostly valued by health workers working in the community (i.e. CHOs). Other cadres of health professionals, including nurses, midwives and district health sisters (DHSs), also reported community service as a motivational factor, but these were in rural postings. It is safe to assume that in these settings their work entails major interaction with the communities. The relationship with the community was a demotivating factor for the same group of health workers (those working directly in the communities).

More variation was seen in the responses of the different cadres of health workers with regard to the reported demotivational factors. This was particularly centred around training opportunities and lack of career progression. Training opportunities, both long and short term, were on offer within the African region or outside the Africa region. However, selection of recipients of training opportunities was reported to be ‘haphazard’, with little thought as to the needs of individual staff and in general focused on mid- to high-level cadre of health professionals. Mid-level cadre health workers were mostly offered on-the-job training. A demotivational factor that pertained mostly to CHOs was the lack of a defined career progression pathway.

With regard to poor management, it is expressed differently, depending on the cadre of health professional. In general, mid-level health professionals felt that their immediate supervisors in the health facilities could be more supportive. Health professionals in supervisory positions, on the other hand, reported having to deal with interference from above that impedes them from executing their administrative and managerial duties in the health facilities effectively and sometimes reported on a perceived breakdown in hierarchy. The support needed from the administrative wing of the MOHS is sometimes weak, and together with political interference, this creates a ripple effect that results in indiscipline in the work place [24] and contributes to health worker shortages in underserved areas, undermines managerial structures and weakens accountability [36]. Political interference has been described in the literature as incompatible with professional ethics and equitable and effective healthcare delivery [37].

Limited financial incentives and benefits was expressed as a demotivational factor by more mid-level cadres of health professionals (e.g. nurses, CHOs, midwives) compared to high-level health professionals (i.e. physicians). The high-level cadre of health professional expressed this as a demotivator in the context of the pre-conflict phase [24], perhaps reflecting recent gains by higher level staff.

### Understanding differences across areas

Of the 17 respondents that reported poor working conditions as a demotivating factor, 11 were currently in rural postings. Working conditions as a demotivating factor can be divided into a number of other subfactors. These include issues pertaining to poor infrastructure in the health facilities, lack of available suitable accommodation or housing allowances and lack of transportation or transport allowance despite working in difficult terrains. Rural health facilities have a number of infrastructural challenges ranging from lack of available space to run functional health facilities, lack of vital utilities to run the facilities and lack of basic and life-saving equipment. This creates a very demanding working environment for the rural health workers compared to urban-posted health workers. Urban health workers also reported, with less frequency compared to rural health workers, having to improvise on the job.

More urban health workers reported poor management and political interference as a demotivating factor compared to rural health professionals. This could be due to more senior health workers with additional administrative duties being included from the Western Area, compared to those from the rural areas. However, the rural-posted health workers also indicate the lack of a support system from their immediate bosses and from a national point of view, with an underlying sense of them feeling detached from the MOHS. In general, health workers posted to rural health facilities take up positions of leadership but are not supported or trained with the required skills to take up managerial roles. This situation is exacerbated by the lack of available human resource for health.

Limited professional training opportunities emerged as a demotivating factor with regional disparities (10 out of the 12 respondents were working in rural areas). In general, the health workers in rural health facilities were of the opinion that training opportunities were only made available to those working in the capital city, Freetown, while in urban areas the complaint was about fairness of allocation rather than the absence of training opportunities per se.

While urban health workers complained more about inflation in the cost of living, and its relationship to salary, rural health workers also felt disadvantaged on the issue of financial incentives. Incentives meant to motivate health workers working in remote areas were reported as not forthcoming, meaning that there is not much difference in the monthly income of health workers working in remote areas and those working in urban areas. At the same time, they face real additional costs, such as travelling to see their families periodically and communicating with them. Moreover, rural-posted health workers are usually overburdened with work and have to work very long hours, especially in light of the introduction of the FHCI, which gives free access to healthcare at the point of delivery for pregnant women, lactating mothers and children under the age of five. This and local market conditions reduce their ability to supplement their salary from other sources of work.

Other demotivating factors such as difficult relationships with the communities, separation from family, personal insecurity and transport challenges were experienced predominately by rural health workers, as they interact more with the communities.

When asked in the ReBUILD survey in an open question about what would motivate them to serve in rural areas, financial incentives were most often cited as important across the whole group (80%), followed by better accommodation (64%), transport support (56%), improved working conditions (18%) and access to basic amenities (14%) [20]. This is in line with the qualitative findings in this article.

### Differences across gender

Gender differences were also captured in this study. Separation from family was predominately expressed by female health workers in rural postings. These health workers based in the hard-to-reach areas are faced with a number of constraints on the job, such as difficult terrain, bad roads, poor communication, delayed allowances or no allowances, while having to deal with an additional emotional burden of being separated from their families. This poses an emotional strain on these female health workers who in addition also voice personal security as a demotivating factor. Given the predominance of women, especially in the low- and mid-level cadres, it is especially important to implement gender-sensitive HR policies.

### Setting the findings in a historical context

These challenges of availability, retention, recruitment, deployment and performance of the health workforce are long-standing [38, 39]. HRH reforms implemented effectively are linked to health worker-improved health system performance and delivery of care [40, 41]. A number of HRH reforms were proposed to address these challenges, which were rarely implemented and were reportedly not driven by evidence. Official HRH documents developed in the post-conflict period, 2002 to 2009, highlighted similar challenges and described similar potential interventions but failed to propose actual implementation plans for the interventions [5]. In addition, most of these policies were driven externally, were fragmented due to lack of coordination between the key players within the health sector and therefore lacked national ownership that would ensure their sustainability. A number of the recommendations coming from staff in this study are already captured in existing HRH policies but the challenge lies in operationalising them, which highlights a bigger system problem. Implementation of HRH reforms became more operational and coherent between 2009 and 2010, as budgeted plans and expenditure frameworks began to emerge with the onset of the ‘window of opportunity’ for major reform in the form of the FHCI in 2010. This brought about a number of HRH reforms and changed the incentive conditions for health workers with the introduction of a number of incentive policies [42, 43]. The lessons of that phase need to be learned in the post-Ebola period, which needs to see a similar combination of political momentum, donor coordination and support and technical assistance to implement the package of measures legitimately required by the health workers.

## Conclusions

Sierra Leone faces a chronic shortage of health workers and also, in common with many other low- and middle-income countries, a concentration of staff in urban centres. The current Ebola outbreak has further exacerbated the substantial health system and human resource challenges. As we move into the post-Ebola reconstructive phase, this study provides important insights into the motivation of health workers and can contribute towards building a resilient and responsive health system, while taking into account the priorities and needs of health workers. This could potentially bridge the gap of mistrust caused by the Ebola epidemic between health workers and service users and could increase the progress towards universal health coverage, particularly in rural areas.

While recent policy reforms have focussed on improving financial conditions, especially for higher level health workers, other non-financial factors now need careful attention. Some demotivating factors, such as political interference in posting and management, appear to have worsened in recent years. Some differing emphases by location, cadre and gender are brought out here. The current centralised recruitment and posting system does not allow for responsiveness. In addition, health workers must be integrated in the process of setting national goals, designing strategies and implementing effective policies and programmes. All these efforts should be directed towards building national capacity, with adequate political backing and effective use of the resources which the post-Ebola window is bringing.

## Appendix

**Table 2:** Study participant characteristics

Characteristic	Average	Range
Age	49 years	36–65 years
Time spent working in the region	20 years	11–35 years
Sex	48% male; 52% female	
Cadres	Medical officers (13%); nurses (22%); midwives (30%) Community Health Officers (35%)	
District	9% Western Area (Rural); 39% Western Area Urban; 13% Bonthe (rural and hard to reach); 22% Koinadugu (rural and hard to reach); 17% Kenema (rural)	
Type of health facility	Tertiary (52%); secondary (17%); primary (30%)

**Table 3:** Coding framework

Themes	Subthemes
1. Decision to join the medical profession	1.1 Individuals who influenced decision to become a health worker (personal, family or other) 1.2 Factors influencing decision to become a health worker ᅟ1.2.1 Respect for the profession in society ᅟ 1.2.2 Availability of funding ᅟ1.2.3 Other
2. Training	2.1 Initial training ᅟᅟᅟ 2.1.1 Location of training ᅟᅟᅟ2.1.2 Length of initial training ᅟ ᅟᅟ2.1.3 Source of funding ᅟᅟ ᅟ2.1.4 Experience of training ᅟᅟᅟ 2.1.5 Reasons for returning home 2.2 Subsequent training/upgrading ᅟᅟᅟ2.2.1 Reasons for subsequent training ᅟ ᅟᅟ2.2.2 Location(s) for subsequent training ᅟᅟ ᅟ2.2.3 Source of funding ᅟᅟᅟ 2.2.4 Length of subsequent ᅟᅟᅟ2.2.5 Experience of subsequent training ᅟ ᅟᅟ2.2.6 Reasons for returning to work to home after the training
3. Career trajectory	3.1 Job description 3.2 Reason for job change ᅟ3.3.1 Self-develop ᅟ3.3.2 Posting ᅟ 3.3.3 Conflict ᅟ3.3.4 Family reasons 3.3 Future career plans ᅟ 3.3.1 Reasons for career plan
4. Overall perception of career	4.1 Satisfaction 4.2 Dissatisfaction 4.3 Motivating factors ᅟ4.3.1 Religion ᅟ 4.3.2 Community service ᅟ4.3.3 Satisfaction about role ᅟ 4.3.4 Financial incentives (salary and allowances) ᅟ4.3.5 Working conditions ᅟ4.3.6 Level of autonomy 4.4 Demotivating factors ᅟ 4.4.1 Professional relationships (with colleagues and superiors) ᅟ4.4.2 Level of autonomy ᅟ4.4.3 Political interference ᅟ 4.4.4 Relationship with communities ᅟ4.4.5 Security ᅟ 4.4.6 Separation of family ᅟ 4.4.7 Limited opportunities for training (regional disparities)
5. International migration	5.1 Factors for migrating 5.2 Reasons for coming back
6. Context of conflict	6.1 Pre-conflict ᅟ 6.1.1 Health workers ᅟ 6.1.2 Health system ᅟ 6.1.3 Coping strategies 6.2 Situation during the conflict ᅟ 6.2.1 Effect on health workers ᅟ 6.2.2 Effect on health system ᅟ 6.2.3 Coping strategies 6.3 Post-conflict situation ᅟ 6.3.1 Health workers ᅟ 6.3.2 Health system ᅟ 6.3.3 Coping strategies
7. Sources of income	7.1 Salary 7.2 Allowances (PBF, RAA) 7.3 User fees 7.4 Gifts from patients 7.5 Private business 7.6 Other sources of income
8. Experiences and perception of Incentive policies	8.1 Incentive policies experienced/heard of 8.2 FHCI ᅟ 8.2.1 Personal effects ᅟ 8.2.2 Health system effects 8.3 RAA ᅟ 8.3.1 Personal effects ᅟ 8.3.2 Health system effects 8.4 PBF ᅟ 8.4.1 Personal effects ᅟ 8.4.2 Health system effects 8.5 Salary uplift ᅟ 8.5.1 Personal effects ᅟ 8.5.2 Health system effects 8.6 Other ᅟ 8.6.1 Personal effects ᅟ 8.6.2 Health system effects
9. Recommendations for a retention package for rural and hard-to-reach areas	
10. General recommendation	
